# MPC1 deficiency accelerates lung adenocarcinoma progression through the STAT3 pathway

**DOI:** 10.1038/s41419-019-1324-8

**Published:** 2019-02-15

**Authors:** Hongbo Zou, Qian Chen, Anmei Zhang, Songtao Wang, Hong Wu, Ye Yuan, Shuang Wang, Jing Yu, Mao Luo, Xianmei Wen, Wei Cui, Wenjuan Fu, Ruilian Yu, Lin Chen, Ming Zhang, Haitao Lan, Xia Zhang, Qichao Xie, Guoxiang Jin, Chuan Xu

**Affiliations:** 1 Institute of Pathology and Southwest Cancer Center, Southwest Hospital, Third Military Medical University (Army Medical University), and Key Laboratory of Tumor Immunopathology, Ministry of Education of China, Chongqing, China; 2 Department of Oncology, Sichuan Academy of Medical Sciences, Sichuan Provincial People’s Hospital, University of Electronic Science and Technology of China, Chengdu, China; 3 Department of Oncology, Third Affiliated Hospital of Chongqing Medical University, Chongqing, China; 4 Department of Oncology, Xinqiao Hospital, Third Military Medical University (Army Medical University), Chongqing, China; 5 Department of Oncology, Chengdu Military General Hospital, Chengdu, China; 6 Department of Oncology, The Affiliated Hospital of Southwest Medical University, Luzhou, China; 7 Department of Dermatology, Chongqing Yubei District People’s Hospital, Chongqing, China; 8 School of Life Science and Biopharmaceutics, Shenyang Pharmaceutical University, Shenyang, China

## Abstract

Mitochondrial pyruvate carrier 1 (MPC1), a key factor that controls pyruvate transportation in the mitochondria, is known to be frequently dysregulated in tumor initiation and progression. However, the clinical relevance and potential molecular mechanisms of MPC1 in lung adenocarcinoma (LAC) progression remain to be illustrated. Herein, MPC1 was lowly expressed in LAC tissues and significantly associated with favorable survival of patients with LAC. Functionally, MPC1 markedly suppressed stemness, invasion, and migration in vitro and spreading growth of LAC cells in vivo. Further study revealed that MPC1 could interact with mitochondrial signal transducer and activator of transcription 3 (mito-STAT3), disrupting the distribution of STAT3 and reducing cytoplasmic signal transducer and activator of transcription 3 (cyto-STAT3) as well as its phosphorylation, while the activation of cyto-STAT3 by IL-6 reversed the attenuated malignant progression in MPC1-overexpression LAC cells. Collectively, we reveal that MPC1/STAT3 axis plays an important role in the progression of LAC, and our work may promote the development of new therapeutic strategies for LAC.

## Introduction

Lung adenocarcinoma (LAC) represents about 40% of overall lung cancers and the leading cause of cancer-related deaths worldwide^1^. Stemness, invasion, and subsequent metastasis, which increase the incidence of recurrence and treatment failure, are the major causes of LAC-related death^2,3^. A better understanding of the molecular mechanisms underlying LAC cell progression is crucial for developing effective treatments.

Aberrant mitochondrial pyruvate metabolism is the important feature in cancer cells, and key enzymes associated with mitochondrial pyruvate metabolism are crucial in the tumor progression^4–6^. Mitochondrial pyruvate carrier 1 (MPC1), which is located in the inner mitochondrial membrane, is one of the key enzymes responsible for pyruvate transportation and oxidation^7–9^, MPC1 deficiency or inactivation accelerates aerobic glycolysis and malignant progression in diverse types of cancer, such as colon cancer and esophageal squamous cell carcinomas^6,10^. Additionally, decreased expression of MPC1 is associated with poor prognosis in various types of tumors, including prostate cancer and colon cancer^11,12^. Collectively, these findings indicate that MPC1 probably serves as a tumor suppressor to impair tumor malignancy.

Despite the involvement of MPC1 in tumor progression, the clinical relevance and function of MPC1 in LAC remains to be investigated. In this study, we showed that MPC1 in LAC was positively correlated with overall survival of LAC patients. Functionally, overexpression of MPC1 attenuated the stemness, invasion, and migration capacities of LAC cancer cells in vitro and in vivo, while knockdown of MPC1 or using MPC inhibitor UK5099 promoted those malignant phenotypes. Mechanically, MPC1 suppressed tumor progression via interacting with mito-STAT3, disrupting STAT3 distribution and inhibiting cyto-STAT3 activation. Thus, our data revealed a critical role of MPC1 in controlling the progression of LAC and its potential therapeutic and prognostic value for LAC patients.

## Materials and methods

### Cell culture and reagents

Human LAC cell lines A549, H1299, H1975, and human normal bronchial epithelial (HBE) cells were purchased from the ATCC (Manassas, VA, USA) and cultured in Dulbecco’s modified Eagle’s medium (DMEM) (HyClone, Logan, UT, USA) supplemented with 10% fetal bovine serum (FBS) (HyClone, Logan, UT, USA) and 100 U/ml penicillin–streptomycin (HyClone, Logan, UT, USA). The cells were maintained in a humidified 37 °C incubator with a 5% CO_2_ atmosphere. UK5099 (Sigma-Aldrich, St. Louis, MO, USA), a MPC1 inhibitor, cells were treated with UK5099 with 40 μM for 48 h^6^. IL-6 (PeproTech, Rocky Hill, NJ, USA), a STAT3 specific agonist, cells were treated with indicated dose for 24 h.

### Patients and tissue specimen

Tumor and adjacent normal tissues from LAC patients who underwent surgical resection in Southwest Hospital, Third Military Medical University (Army Medical University) from May 2016 to June 2017, Chongqing, China, with approval from the Institutional Ethics Committee. The tissue microarray, including 78 LAC without radiotherapy or chemotherapy before surgery, was from OUTDO BIOTECH (Shanghai, China). All patients were provided with informed consent and clinical data were listed in Supplementary Table 1.

### Immunohistochemical (IHC) staining and scoring

Human Tissue slices were deparaffinized and hydrated by a series of xylene and alcohol treatment, and the procedure was performed as previously described^13^. The slices were incubated with rabbit polyclonal anti-MPC1 (1:250; Abcam, Cambridge, UK) and anti-P-STAT3(Y705) (1:100; Abcam, Cambridge, UK); anti-SOX2 (1:100; CST, Danvers, MA, USA); anti-MMP2 (1:100; CST, Danvers, MA, USA) antibodies at 4 °C overnight, followed by incubation with avidin–biotin–peroxidase (DAKO). MPC1 expression using the following system: 0, 0–5% positive cells; 1, < 25% positive cells; 2, 25–50% positive cells; 3, 50–75% positive cells; and 4, 75–100% positive cells. The staining intensity was scored as follows: 0, no positive staining; 1, weak staining; 2, moderate staining; 3, strong staining. The final scores were obtained by multiplying the extent scores by intensity scores and analysis using the statistical X-tile software with score of 4 as the cutoff value^14^.

### GEO database analysis

A total number of 1926 tumor samples were used to generate Kaplan–Meier curves for the survival analysis of MPC1 in LAC (http://www.kmplot.com/lung). The median expression was used as the final cutoff value.

### Lentiviral infection procedures

For construction of the MPC1 overexpressing cell line, Flag (EF-1a/Puro/Amp) lentiviral vector (Hanbio, shanghai, China) containing human MPC1 was used to transfect cancer cells. Lentiviral particles packaged with a blank vector were used as a negative control. A549 and H1299 cells (1 × 10^6^/well) were infected with lentivirus containing Ctrl or MPC1-Flag. Cells were selected and enriched by 4 μg/ml puromycin. For the si*MPC1* cell line, H1299 cells (1 × 10^6^/well) were infected with siRNAs targeting MPC1 (GenePharma, Shanghai, China). Cell transfection was performed using Lipofectamine 2000 (Invitrogen, Carlsbad, CA, USA). The siRNAs were list as follows, MPC1 siRNAs were custom synthesized from GenePharma (Shanghai, China) and were pooled as (5′-UCCCTCTUTTUCTATTCTTTU-3′) and (5′-UUCTTATCAAACACUAUATUA-3′).

### Quantitative real-time PCR (qRT-PCR)

Total RNA was extracted from cancer cells with RNAiso reagent according to the manufacturer’s protocol. Reverse transcription and qRT-PCR were performed using a One Step SYBR primer. MPC1: forward: 5′-TGGCTAAAGGAGCAGAGGAA-3′, reverse: 5′-ATGACCACATCACGGCTACA-3′ GAPDH: forward: 5′-TGTTCGTCATGGGTGTGAAC-3′, reverse: 5′-ATGGCATGGACTGTGGTCAT-3′.

### Western blotting

Western blotting was performed as previously described^15^. The primary antibodies were as follows: anti-MPC1(1:1000; Abcam, Cambridge, UK); anti-GAPDH (1:1000 CST, Danvers, MA, USA); anti-COX IV (1:1000 CST, Danvers, MA, USA); anti-β-actin (1:1000 CST, Danvers, MA, USA); anti-P-STAT3 (Y705)(1:1000; CST, Danvers, MA, USA); anti-P-STAT3 (S727) (1:1000; CST, Danvers, MA, USA); anti-STAT3 (1:1000; CST, Danvers, MA, USA); anti-OCT4 (1:1000 CST, Danvers, MA, USA); anti-MMP2 (1:1000 CST, Danvers, MA, USA); anti-MMP3 (1:1000 CST, Danvers, MA, USA); anti-MMP7 (1:1000 CST, Danvers, MA, USA); anti-SOX2 (1:1000; CST, Danvers, MA, USA); anti-NANOG (1:1000; CST, Danvers, MA, USA).

### Nuclear and cytoplasmic extraction assays

The NE-PER Nuclear and Cytoplasmic Extraction Reagents (Thermo Scientific, Waltham, MA, USA) were purchased from Thermo Scientific. The detailed procedures were performed according to the manufacturer’s instructions.

### Isolation of mitochondrion and cytoplasm fractions

Mitochondrion and cytoplasm fractions from tumor cells were separated using Mitochondria Isolation Kit (Solarbio, Beijing, China) (number: SM0020) following the manufacturer’s protocol.

### Co-immunoprecipitation (Co-IP) and mass spectrometry (MS) analyses

For Co-IP, LAC cells were infected with MPC1 overexpressing lentivirus or control lentivirus, which contained a 3 × flag sequence. Anti-flag M2 Magnetic Beads (Sigma-Aldrich, St. Louis, MO, USA) were used for detection and capture of the fusion proteins. The detailed procedures were performed according to the manufacturer’s instructions. For the liquid chromatography–MS analysis, the gel pieces with MPC1 immunoprecipitated complex or control were dehydrated in acetonitrile, dried in a speed vacuum, and digested with trypsin. The peptides were extracted from the polyacrylamide and were evaporated for MS analysis using LTQ-Orbitrap Elite Mass Spectrometer System (Thermo Scientific, Waltham, MA, USA).

### Tumorsphere-formation assay

Cancer cell were seeded into 96-well plates (1 × 10^2^/well) by flow cytometry (Beckman Coulter, S. Kraemer Boulevard Brea, CA, USA). Cells were cultured in DMEM/F12 stem cell medium containing basic fibroblast growth factor (bFGF), epidermal growth factor (EGF), and B-27 for 1 week, the tumorspheres were photographed by a light microscope and counted to calculate the tumorsphere efficiency.

### Tumor cell invasion and migration assay

Tumor cell invasion assay was performed as previously described^13^. For the migration assay, 1 × 10^4^ cells were added to the transwell chambers with 8-μm pore size (Millipore, Billerica, MA, USA). After incubation for 24 h, the chambers were wiped using cotton swabs, fixed with 4% paraformaldehyde, and stained with crystal violet. The lower chamber was photographed using a light microscope, and the cells were counted.

### Invadopodia immunofluorescence staining

Tumor cells grown on glass coverslips were fixed with 4% paraformaldehyde for 10 min, permeabilized by 0.3% Triton X-100 for 5 min, and then incubated with anti-rhodamine phalloidin (Solarbio, Beijing, China). Cells were then photographed under a laser confocal scanning microscope (Leica, Brunswick, Ohio, Germany). Cells with more than four microspikes on the surface were considered invadopodia-positive cells^16^. The cells were counted in 10 separate, randomly selected 200 × fields with a fluorescence microscopy.

### Xenograft

Four-week-old female BALB/c athymic nude mice were approved by the Institutional Animal Care and Use Committee of the Southwest Hospital, TMMU. The number of 1 × 10^6^ MPC1-overexpression cells or control cells were suspended in 50 μl of PBS and subcutaneously implanted in female nude mice. The size of the xenograft tumor was measured every 4 days for a month using a Vernier calliper, and the volume was calculated at different indicated intervals post-transplantation as follows: volume = shortest diameter^2^ × longest diameter/2. The animals were killed 1 month after cell implantation, and the subcutaneous xenografts were weighed and analyzed by IHC. The animal experiments were approved by the Institutional Animal Care and Use Committee of Southwest Hospital, Third Military Medical University in accordance with the Guide for the Care and Use of Laboratory Animals.

### Statistical analysis

All experiments were performed at least three times with triplicate samples. Data were analyzed using SPSS 16.0 software (SPSS, Chicago, IL, USA) and GraphPad Prism 6.0 (San Diego, CA, USA) and were expressed as the mean ± SD. Unless otherwise noted, the data were analyzed using Student’s *t* test or one-way ANOVA. Significance of correlation between the expression of MPC1 and histopathological factors were determined using Pearson’s χ^2^ test. Kaplan–Meier plots were performed to investigate the prognostic relevance of MPC1 in univariate analysis. Multivariate analysis was performed by applying Cox proportional hazards test. Statistical difference was considered significant if *P*-values were < 0.05. All significant statistical differences were defined as *P<0.05; **P<0.01, and ***P<0.001.

## Results

### MPC1 is downregulated in LAC and predicts favorable prognosis

**Fig. 1:**
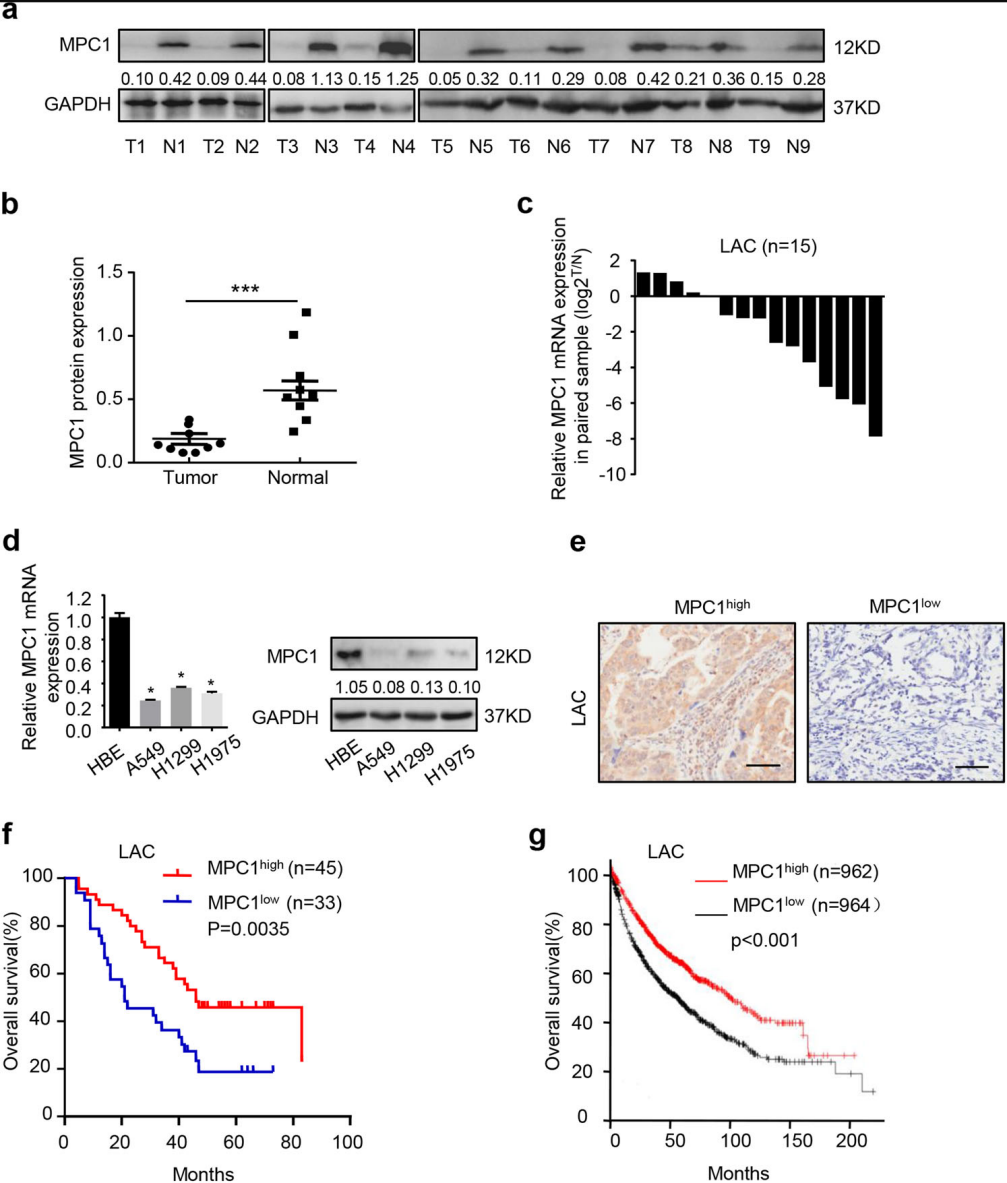
MPC1 is downregulated in human LAC and predicts good prognosis. **a** Western blotting showing the expression of MPC1 in nine pairs LAC tumor tissue (T) and adjacent normal tissues (N). **b** Quantitative analysis of MPC1 protein expression in nine pairs LAC tumor tissue and adjacent normal tissues. **c** Expression of MPC1 mRNA in 15 pairs of LAC tumor tissues and adjacent normal tissues. **d** qRT-PCR and western blotting showing the expression of MPC1 in LAC cells (A549, H1299, H1975) and normal human bronchial epithelial cells (HBE). **e** Representative IHC image for LAC patients with MPC1^low^ and MPC1^high^, Scare bar = 50 μm. **f** Kaplan–Meier analysis of overall survival rate (OS) in LAC patients with MPC1^low^ and MPC1^high^. **g** Kaplan–Meier analyses of MPC1 in LAC patients from GEO database (*n* = 1926). Data are expressed as the mean ± SD,*P<0.05, ***P<0.001

To determine the status of MPC1 in LAC, we examined the expression of MPC1 in nine pairs of LAC tissues, and found that MPC1 was lowly expressed in LAC tissues as compared with corresponding adjacent non-tumor tissues (Fig. 1a, b). Furthermore, MPC1 mRNA also decreased in LAC tissues as compared with that in corresponding normal tissue (Fig. 1c), and the similar results were observed in LAC cells (A549, H1299, H1975) as compared with normal human bronchial epithelial (HBE) cells (Fig. 1d). Additionally MPC1 was negatively correlated with tumor size and TNM stages, but not related with tumor location, histological grade, EGFR mutation (Supplementary Table 2). Furthermore, the overall survivals (OS) of LAC patients with MPC1^low^ showed shorter than that with MPC1^high^ in our clinical cohort (Fig. 1e, f) and GEO database (Fig. 1g). Univariate and multivariate analyses further indicated that MPC1 was an independent prognostic indicator for OS of LAC patients (Table 1). Therefore, MPC1 might act as an indicator of favorable prognosis for patients with LAC.

**Table 1:** Univariate and multivariate analysis for overall survival in LAC patients

Factors	Univariate		Multivariate	
	HR (95% CI)	*P-*value	HR (95% CI)	*P-*value
Gender	0.591 (0.337–1.038)	0.067	0.484 (0.267–0.874)	0.016
Age	1.030 (0.992–1.069)	0.992	1.584 (0.863–2.905)	0.137
Location	0.908 (0.515–1.530)	0.746	0.940 (0.508–1.742)	0.845
MPC1 expression	0.558 (0.318–0.969)	0.038	0.583 (0.327–1.040)	0.048
Grade	1.429 (0.948–2.129)	0.112	2.123 (1.497–3.288)	0.619
TNM Stage	1.833 (1.309–2.567)	0.001	1.760 (1.223–2.531)	0.002

*HR* hazard ratio, *CI* confidence interval, *TNM* tumor lymph node metastasis

### MPC1 deficiency augments the stemness of LAC cells in vitro

**Fig. 2:**
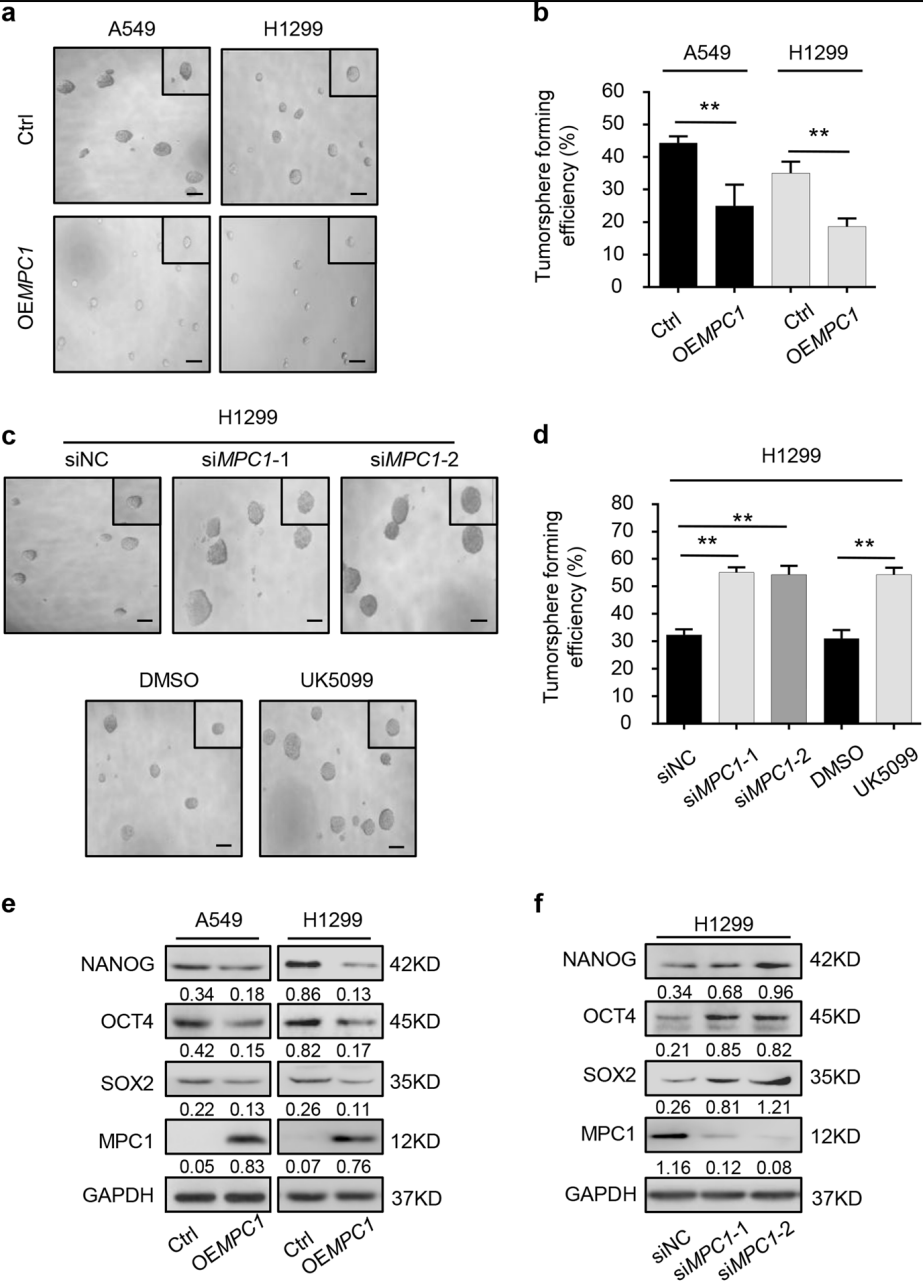
MPC1 deficiency promotes the stem-like traits of LAC cells. **a**, **b** The representative images (**a**) and numbers (**b**) of tumorsphere in OE*MPC1* cells and Ctrl cells by tumorsphere formation assay, scale bar = 100 µm. **c**, **d** The representative images (**c**) and numbers (**d**) of tumorsphere in si*MPC1* and siNC LAC cells, and cells treated with DMSO or UK5099 (40 μM) by tumorsphere formation assay, scale bar = 100 µm. **e**, **f** Western blotting analysis of the abundances of NANOG, OCT4, and SOX2 in up- (**e**) and downregulated (**f**) MPC1 groups as compared with control groups. Data are expressed as the mean ± SD, **P<0.01

To explore the potential relationship between MPC1 status and the stemness of LAC cells, MPC1-overexpression and MPC1-knockdown cells were established (Supplementary Figure 1). The volume and number of tumorspheres derived from OE*MPC1* cells were smaller than those of control cells (Fig. 2a, b), whereas knockdown of MPC1 or inhibiting MPC1 activity treated with UK5099 increased those capabilities (Fig. 2c, d). In addition, western blotting revealed that overexpression of MPC1 decreased the level of cancer stem cell (CSC) markers, including NANOG, OCT4, and SOX2 in LAC cells (Fig. 2e), while knockdown of MPC1 increased the level of these markers (Fig. 2f). Conclusively, these data indicate MPC1 deficiency increased cancer stem-like traits of LAC cells.

### Loss of MPC1 accelerates the migration and invasion of LAC cells in vitro

**Fig. 3:**
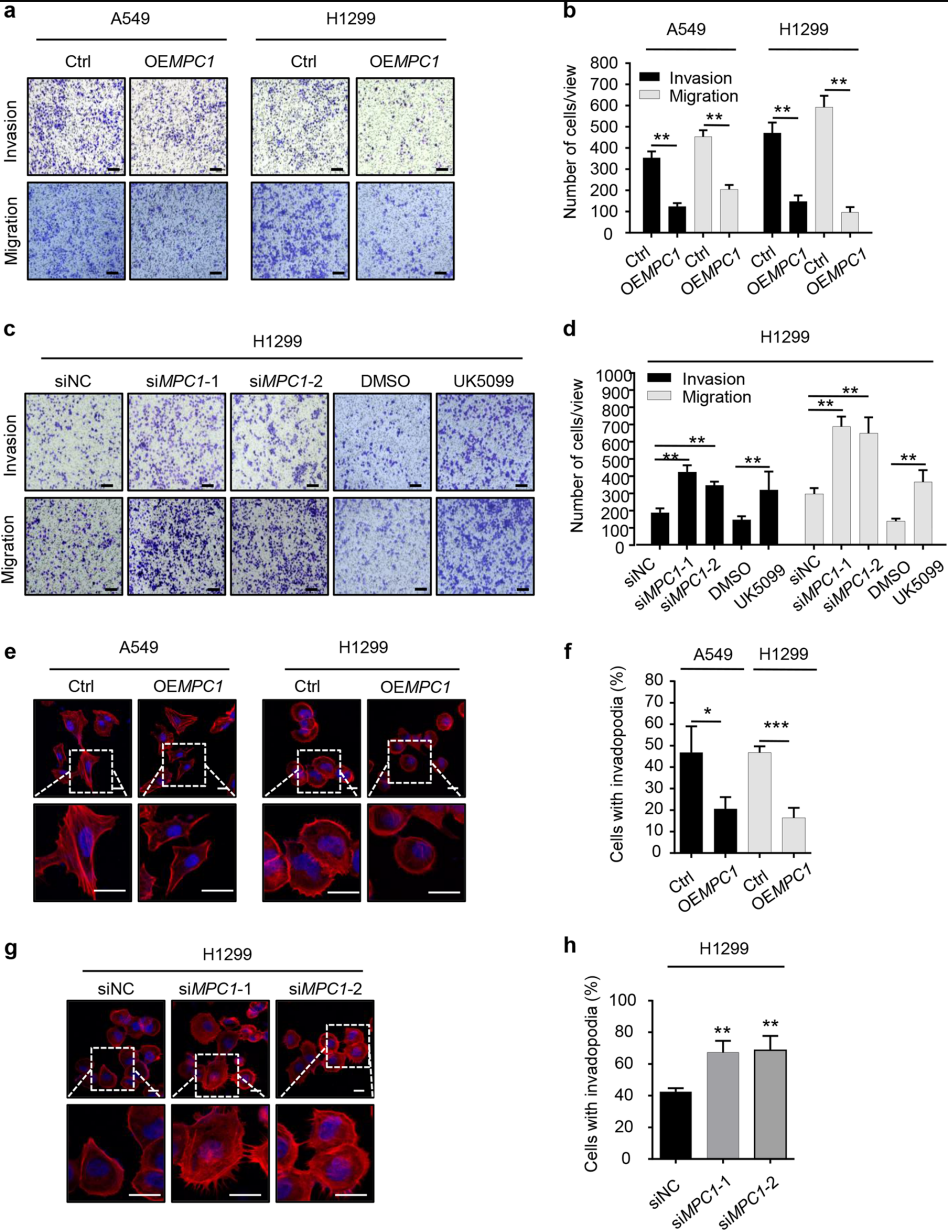
MPC1 attenuates invasion and migration ability of LAC cells. **a**, **b** The representative images (**a**) and numbers (**b**) of motile OE*MPC1* cells and Ctrl cells in the transwell invasion and migration assay (scale bar = 50 µm). **c**, **d** The representative images (**c**) and numbers (**d**) of motile siNC, si*MPC1* in H1299 cells; DMSO- and UK5099-H1299 cells in the transwell invasion and migration assay (scale bar = 50 µm). **e**, **f** Immunofluorescence showing the representative images (**e**) and numbers (**f**) of invadopodia with OE*MPC1* or Ctrl, the nuclei were stained with DAPI (blue) (Scale bar = 20 µm). **g**, **h** Immunofluorescence showing the representative images (**g**) and numbers (**h**) of invadopodia with si*MPC1* or siNC, the nuclei were stained with DAPI (blue) (scale bar = 20 µm), Data are expressed as the mean ± SD, *P<0.05, **P<0.01, ***P<0.001

Next, we determined whether MPC1 can influence the invasion and migration of LAC cells. The transwell assay showed that overexpression of MPC1 significantly reduced motile ability of LAC cells (Fig. 3a, b), while treatment with si*MPC1* or UK5099 in H1299 cells increased their mobility (Fig. 3c, d). Invadopodia, a form of cellular projection that is embedded in or protruding from the lamellipodial actin network, and matrix metalloproteinases (MMPs) are critical in migration and invasion of cancer cells^17,18^. We revealed that the number of invadopodia in the OE*MPC1* LAC cells was decreased (Fig. 3e, f), while in the si*MPC1* H1299 cells, the number of invadopodia was increased (Fig. 3g, h). Additionally, western blotting showed that the expression levels of MMP2, MMP3, and MMP7 were significantly decreased in OE*MPC1* group (Supplementary Figure 2a), while downregulated MPC1 increased the expression levels of those proteins (Supplementary Figure 2b). Collectively, these observations demonstrate that MPC1 decreases the invasion and migration capabilities of LAC cells through invadopodia formation and the MMPs pathway.

### MPC1 interacts with mito-STAT3 to inhibit cyto-STAT3 activation in LAC cells

**Fig. 4:**
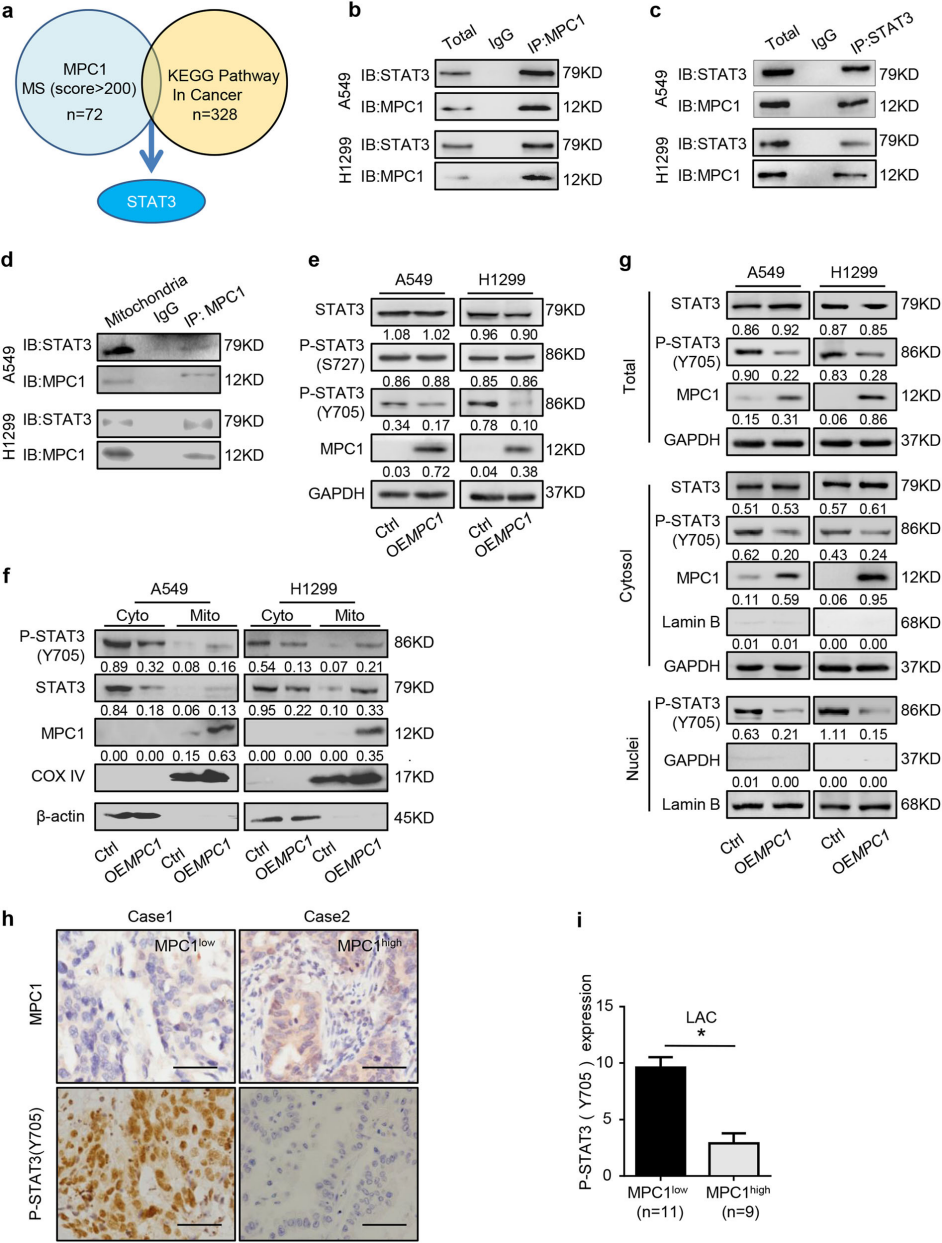
MPC1 interacts with STAT3 in mitochondria and decreases its phosphorylation and transloacation into nuclei. **a** Identification of STAT3 from the intersection between MPC1 interacting protein set by MS and the KEGG pathway in cancer gene set from GSEA. **b**, **c** Co-IP analysis of STAT3 interaction with MPC1 in A549 and H1299 MPC1-overpxpression cells. **d** The mitochondria was isolated from the cytoplasm, and Co-IP analysis showing that STAT3 interacts with MPC1 in the mitochondria in A549 and H1299 MPC1-overpxpression cells. **e** Western blotting showing that the protein expression levels of STAT3, P-STAT3(S727), P-STAT3(Y705), and MPC1 in A549 and H1299 cell with stable infection of OE*MPC1* or Ctrl vector. **f** The mitochondria was isolated from the cytoplasm, mitochondria (mito) and cytoplasm without mitochondria (cyto) were prepared, western blotting showing that the protein expression levels of P-STAT3(Y705), STAT3, MPC1, and mitochondrial reference COX IV in A549 and H1299 cell with stable infection of OE*MPC1* or Ctrl vector. **g** The nuclear and cytoplasmic extracts were prepared. Western blotting showing that overexpression of MPC1 decreased the expression of P-STAT3(Y705) in the nuclei. **h** IHC showing the expression of P-STAT3(Y705) in the MPC1^low^ LAC tissues (*n* = 11) and MPC1^high^ (*n* = 9) LAC tissues, Scale bar = 50 μm. **i** IHC score of P-STAT3(Y705) in the MPC1^low^ LAC tissues (*n* = 11) and MPC1^high^ LAC tissues (*n* = 9). Data are expressed as the mean ± SD, *P<0.05

To further explore the underlying mechanism of MPC1 in progression of LAC cells, we screened potential MPC1-binding proteins through mass spectrometric (MS) analysis. STAT3 was identified in the intersection candidate of MPC1 interacting protein set with the KEGG pathway in cancer gene set from GSEA (Fig. 4a). Furthermore, Co-IP assays further demonstrated that MPC1 can interact with STAT3 rather than P-STAT3 (Fig. 4b, c and Supplementary Figure 3a). STAT3 is present in mitochondrial (mito-STAT3) and cytoplamic (cyto-STAT3) fractions^19^. To further define the location, where the interaction between MPC1 and STAT3 occured, we extracted the mitochondria from cytoplasm, and the Co-IP assays demonstrated that MPC1 could interact with mito-STAT3 (Fig. 4d), but not cyto-STAT3 (Supplementary Figure 3b). Additionally, overexpression of MPC1 downregulated P-STAT3(Y705), whereas knockdown of MPC1 upregulated P-STAT3(Y705), but without affecting P-STAT3(S727) and total STAT3 expression (Fig. 4e and Supplementary Figure 3c). Since cyto-STAT3 can be recruited into the mitochondrial inner membrane by some chaperone, and MPC1 is located in the inner membrane of mitochondrial, we proposed that the binding of MPC1 with STAT3 in the mitochondria promotes the process that cyto-STAT3 is transported into the mitochondria. To test this possibility, we overexpressed MPC1 expression in two LAC cell lines and determined the protein abundance of STAT3 in mitochondria fractions and in cytoplasm fractions. Western blotting shows that overexpression of MPC1 increased the level of STAT3 anchored on mitochondrion, but decreased the level of STAT3 and P-STAT3(Y705) in cytoplasm without mitochondria (Fig. 4f). Previous study shows that STAT3 promotes cancer progression in various types of cancer and is activated by phosphorylation at Tyr705(Y705)^20,21^, and P-STAT3 is translocated into the nuclei to induce the transcription of several target genes implicated in cancer cell malignancy^22^. Cell fractionation analysis demonstrated that overexpression of MPC1 reduced P-STAT3(Y705) in the cytoplasm and nuclei (Fig. 4g). Furthermore, IHC analysis of both MPC1 and P-STAT3(Y705) in the specimens from 20 LAC patients revealed that P-STAT3(Y705) was highly expressed in the MPC1^low^ group (Fig. 4h, i). Taken together, these data indicate that MPC1 interacts with mito-STAT3 and disrupts the location of STAT3 in the cytoplasm and mitochondria, which decreases P-STAT3(Y705) into the nuclei.

### MPC1/ STAT3 axis regulates the progression of LAC cells

**Fig. 5:**
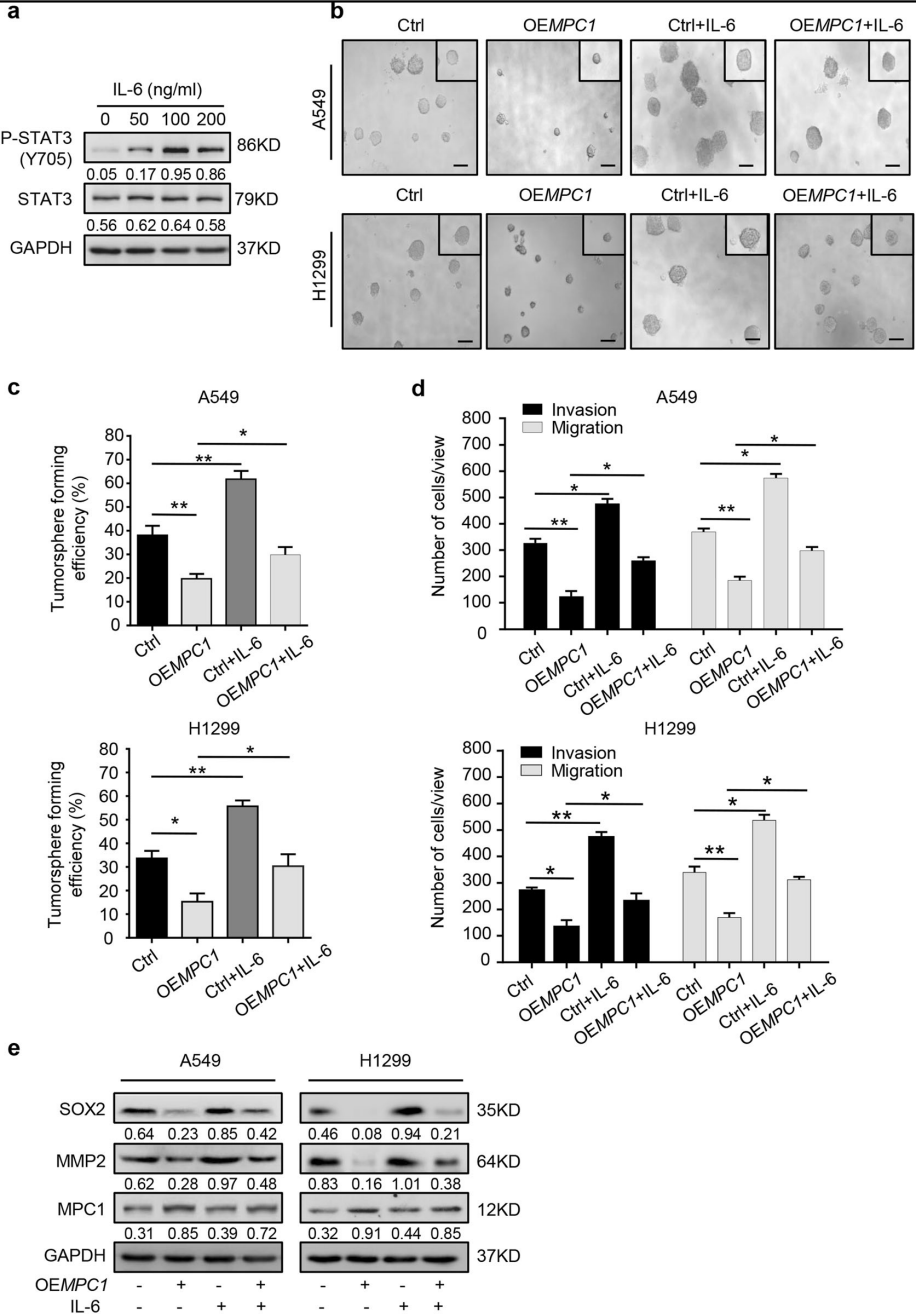
MPC1/STAT3 axis regulates the progression of LAC cells. **a** Western blotting showing the protein expression levels of STAT3 and P-STAT3(Y705) in A549 cells treated with IL-6 for 24 h at the indicated dose. **b**, **c** The representative images (**b**) and numbers (**c**) of tumorspheres from the Ctrl and OE*MPC1* A549 and H1299 cells treated with or without IL-6 (100 ng/ml). Scale bar = 100 µm. **d** The number of motile Ctrl and OE*MPC1* A549 and H1299 cells treated with or without IL-6 (100 ng/ml). **e** Western blotting showing the expression of SOX2, MMP2, and MPC1 in Ctrl or OE*MPC1* A549 and H1299 cells treated with or without IL-6 (100 ng/ml). Data are expressed as the mean ± SD, *P<0.05, **P<0.01

Since MPC1 binds to mito-STAT3 and inhibits the phosphorylation of STAT3, we explored whether MPC1-mediated cancer cell progression depends on the downstream of the STAT3 pathway. Previous report^23^ and our data demonstrated that IL-6 acts as the agonist for the phosphorylation of STAT3 (Fig. 5a). We found OE*MPC1* LAC cells treated with IL-6 (100 ng/ml) increased the stemness (Fig. 5b, c) and mobility capacities (Fig. 5d and Supplementary Figure 4), which partially overcame the inhibitory effects of MPC1 on progression of LAC cells. Furthermore, OE*MPC1* LAC cells treated with IL-6 (100 ng/ml) partially recovered the basal expression levels of SOX2 and MMP2 (Fig. 5e). Altogether, STAT3 was a novel downstream regulator of MPC1 to mediate progression in LAC cells.

### Overexpression of MPC1 inhibits tumorigenicity in vivo

**Fig. 6:**
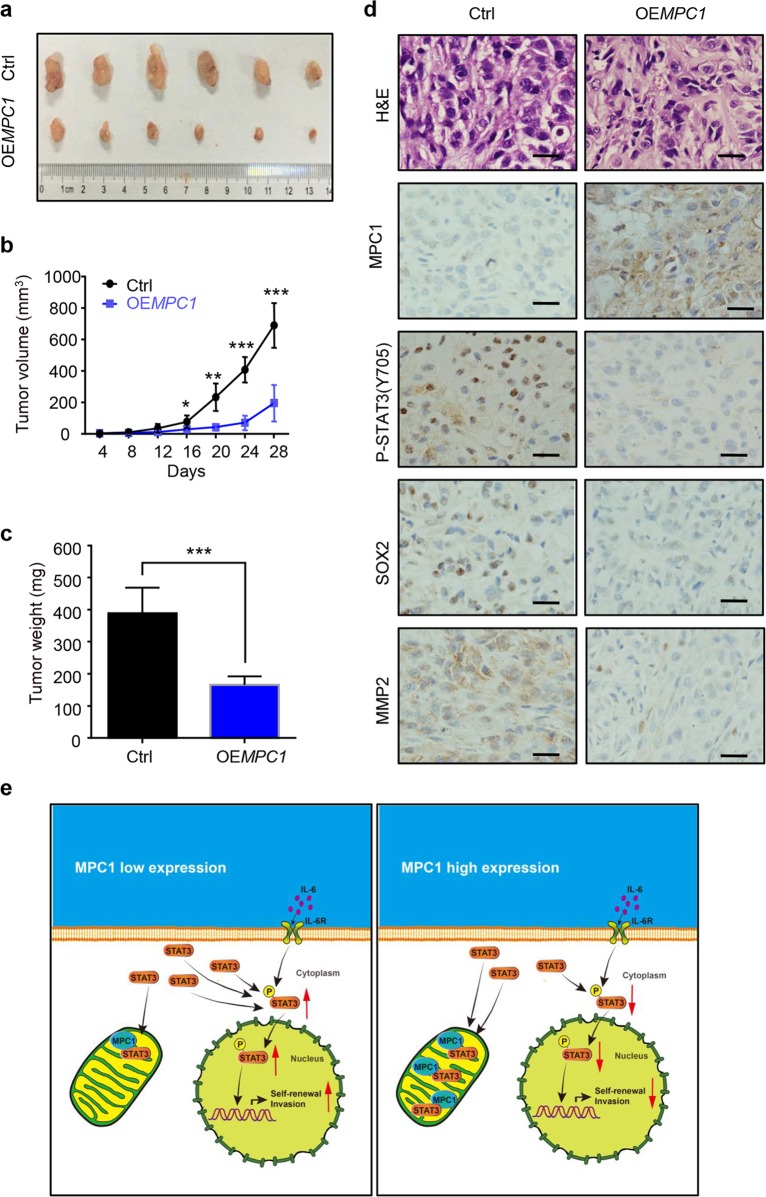
MPC1 suppresses LAC cells xengraft growth in vivo. **a** Nude mice were injected subcutaneously in the right flanks with Ctrl or MPC1-overexpression A549 cells. After 28 days, xenografts were harvested and photographed. **b**, **c** Quantitative analysis of the volume (**b**) and weight (**c**) of the xenograft tumors. **d** IHC staining showing the expression of MPC1, P-STAT3(Y705), SOX2, and MMP2 in the xenograft tumors derived from OE*MPC1* and Ctrl cells, Scale bar = 25 µm. **e** Schematic diagram of the regulatory mechanism of MPC1 mediates STAT3 dephosphorylation and inhibits the stemness and invasion of LAC cells. Data are expressed as the mean ± SD,*P<0.05, **P<0.01, ***P<0.001

To further examine the effect of MPC1 on LAC cells in vivo, the subcutaneous xenograft model was established. The xenograft tumors formed by OE*MPC1* cells had a slower growth rate and smaller average volume as compared with those formed by control cells (Fig. 6a–c). Consistent with our previous data, IHC showed that the expression of P-STAT3(Y705), SOX2, and MMP2 in the OE*MPC1* groups was decreased compared with the control group (Fig. 6d). Therefore, these results indicate that upregulated MPC1 inhibits the tumorigenicity of LAC cells in vivo.

## Discussion

The “Warburg effect” is considered a vital hallmark of cancer cells, characterized by an altered pyruvate metabolism, indicating mitochondrial pyruvate carrier is involved in the progression of cancer^5^. MPC1, which is located at the genomic locus of 6q27 and known as BRP44L, has been reported to form a heterologous protein complex with MPC2 in the inner mitochondrial membrane and acts as a gatekeeper for pyruvate entry into the mitochondria^24,25^. Actually, MPC1 has been reported to be downregulated in several types of cancers, such as gastric cancer^26^ and colon cancer^27^, but the clinical relevance and function of MPC1 in LAC remains elusive. Herein, we revealed that MPC1 was lowly expressed in LAC tissues and positively correlated with prognosis. Further studies showed that the malignancy of LAC cells were increased via depletion of MPC1 or MPC1 inhibitor, but decreased by overexpression of MPC1 in vitro and in vivo. Thus, these data indicate that MPC1 could act as a tumor suppressor and a candidate for therapeutic target as well as a prognostic biomarker in LAC.

Previous studies have shown that MPC1 can transport pyruvate into the mitochondria for oxidation, reducing lactate production in PGC1α-transduced cholangiocarcinoma cells, but elevating reactive oxygen species (ROS) production to facilitate metastatic dissemination of cholangiocarcinoma cells^28^. It has been found that MPC inhibitor UK5099 treatment in esophageal squamous epithelium cells can block pyruvate transportation into the mitochondria so as to attenuate mitochondrial oxidative phosphorylation (OXPHOS) and trigger aerobic glycolysis. In addition, UK5099-treated cells exhibit stronger invasive capacity compared with the parental cells and is more resistant to chemotherapy and radiotherapy. The HIF-1α expression and ROS production were also activated^10^. However, whether MPC1-overexpression could regulate Warburg effect to affect the production of lactate in LAC cells has not been illustrated yet. Our results showed that upregulated MPC1 significantly decreased the production of lactate compared with the control groups (data not shown), suggesting that MPC1 may increase pyruvate into the mitochondria for oxidation and decreased lactate production in LAC cells.

Invasion is correlated with invadopodia formation. Actin cytoskeleton and actin-binding proteins control the dynamic changes in the formation of polarized actin-based migratory protrusions, such as lamellipodia, filopodia, apically restricted circular dorsal ruffles (CDRs), and invadopodia. Herein, we found MPC1 could act as a regulator in invadopodia to control LAC cell invasion.

Our study also revealed that MPC1 was a key inhibitory factor in sustaining the stemness capability of LAC cells by decreasing the expression of stemness biomarker. In our previous work, we have shown that IGF-1R mediated OCT4 expression to form a complex with β-catenin and SOX2, which is crucial for the stemness of LAC cells^13,29^. Despite IGF-1R, our study on MPC1 may bring forward a new underlying mechanism of the stemness traits in LAC.

STAT3, a member of the signal transducer and activator family, was phosphorylated on tyrosin 705 (Y705) and serine 727 (S727) residue to form a dimmer and then translocated into the nuclei upon binding of growth factors and cytokines with their receptors, such as IL-6^30^. P-STAT3 acts as a transcription factors to activate downstream target genes that are implicated in cancer progression^31^. However, robust and stable antitumor STAT3 activity has not been observed in clinical trials^32^. Recent reports showed STAT3 is not only present in the cytoplasm, but also resides in the inner mitochondrial membrane to increases activity of complex I and II of the electron transport chain to regulate cellular respiration in a transcriptional independent manner, and mitochondrial STAT3 sustained glycolytic and oxidative phosphorylation activities of cancer cells^33–35^. Additionally, the mitochondrial STAT3 suppresses autophagy induced by oxidative stress and may effectively preserve the mitochondria from being degraded by mitophagy^36^. Gene associated with retinoid interferon-induced cell mortality 19 (GRIM-19) can recruit STAT3 by the site of S727A into the mitochondria and enhance the integration of STAT3 involved in celluar respiration and glycolysis^37^. These data indicate that altering the balance between cyto-STAT3 and mito-STAT3, leading to the phosphorylation of STAT3 may be an effective way to combat cancer. Our work identified the interaction between mito-STAT3 and MPC1, which preventing STAT3 distribution in the cytoplasm and decreasing its phosphorylation in the cytoplasm and nuclei.

In summary, our study illustrates an important function of MPC1 in controlling the malignant behavior of LAC and acts as a novel prognostic indicator for LAC patients. Furthermore, we identify that MPC1/STAT3 axis is crucial in the progression of LAC (Fig. 6e), which provides a new train of thought regarding the molecular targeting of STAT3 pathway.

## Supplementary information

Supplementary Figure 1

Supplementary Figure 2

Supplementary Figure 3

Supplementary Table 1

Supplementary Table 2

Figure legend
