# Supplementary figures and images for: MPC1 deficiency accelerates lung adenocarcinoma progression through the STAT3 pathway
Source: Cell Death Dis. 2019 Feb 15;10(3):148. doi: 10.1038/s41419-019-1324-8 (PMC6377639; doi:10.1038/s41419-019-1324-8)

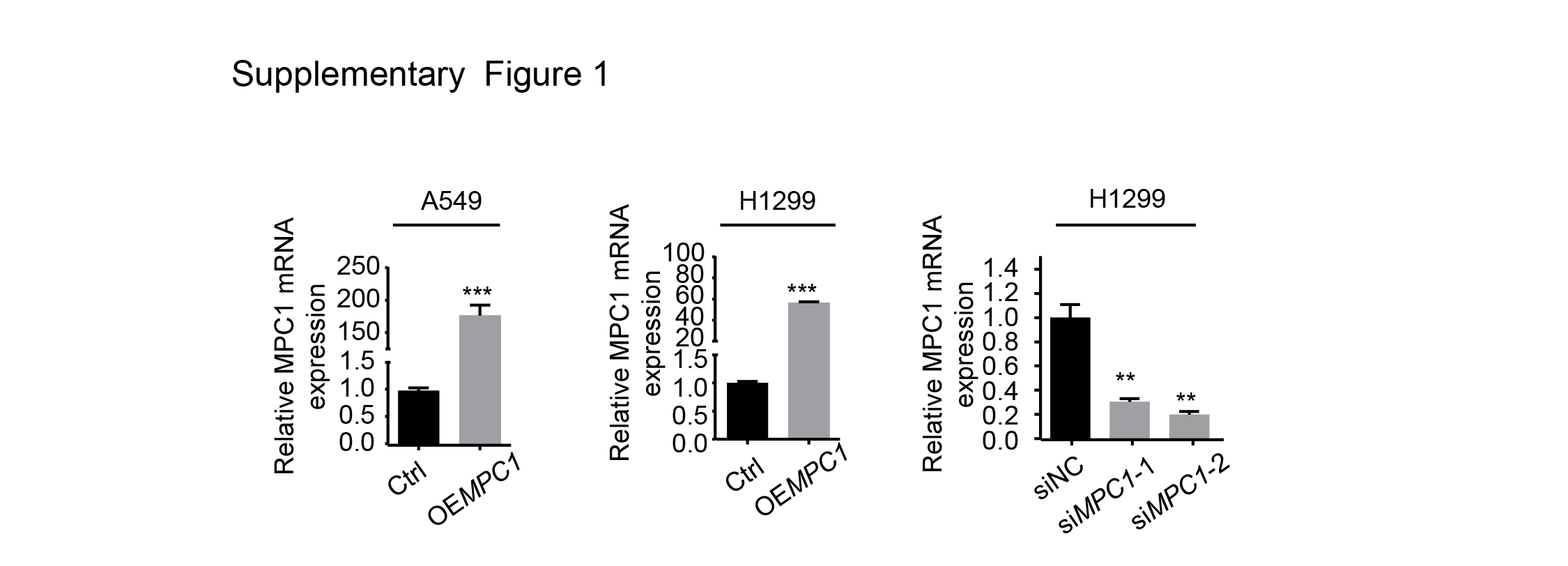

Supplement: Supplementary file 1 — Supplementary Figure 1 [file 41419_2019_1324_MOESM1_ESM.tif]

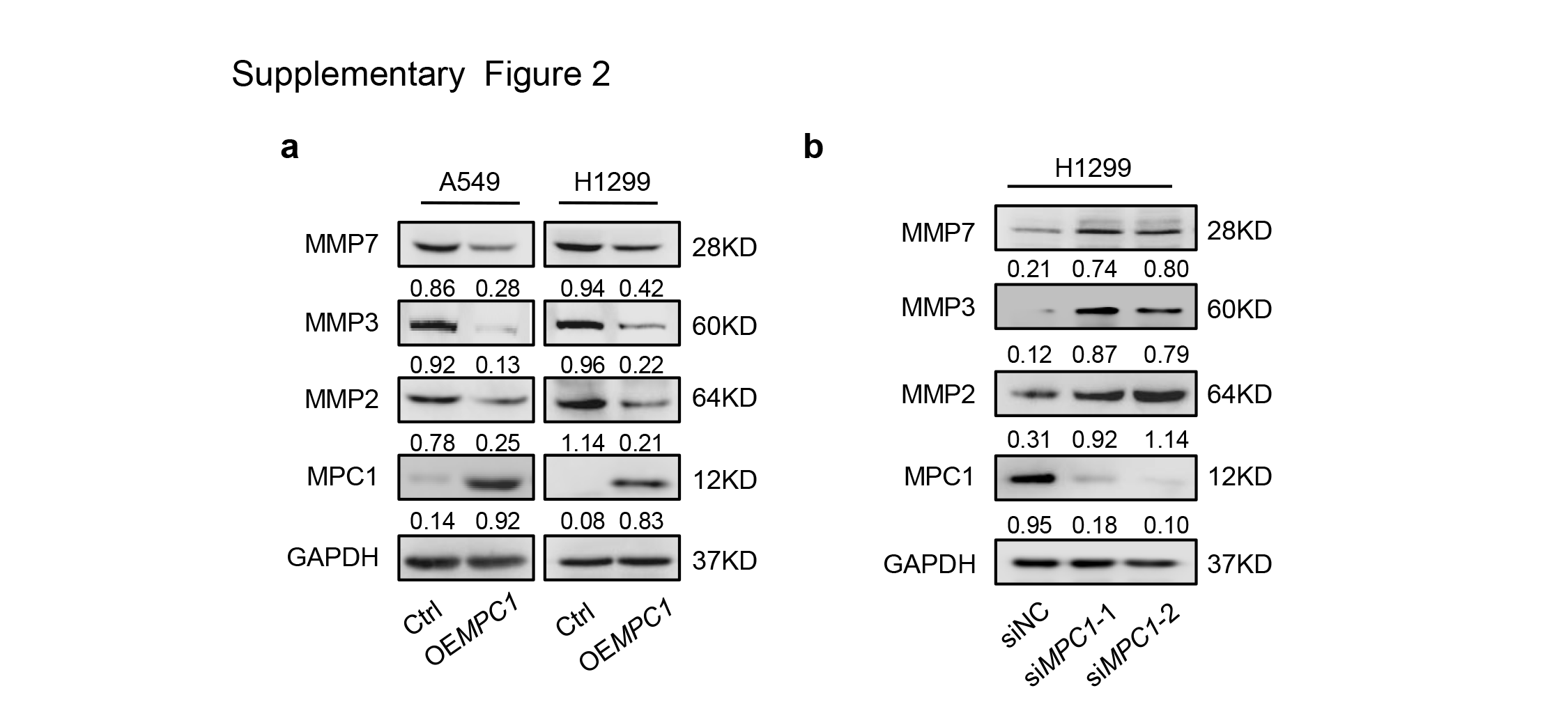

Supplement: Supplementary file 2 — Supplementary Figure 2 [file 41419_2019_1324_MOESM2_ESM.tif]

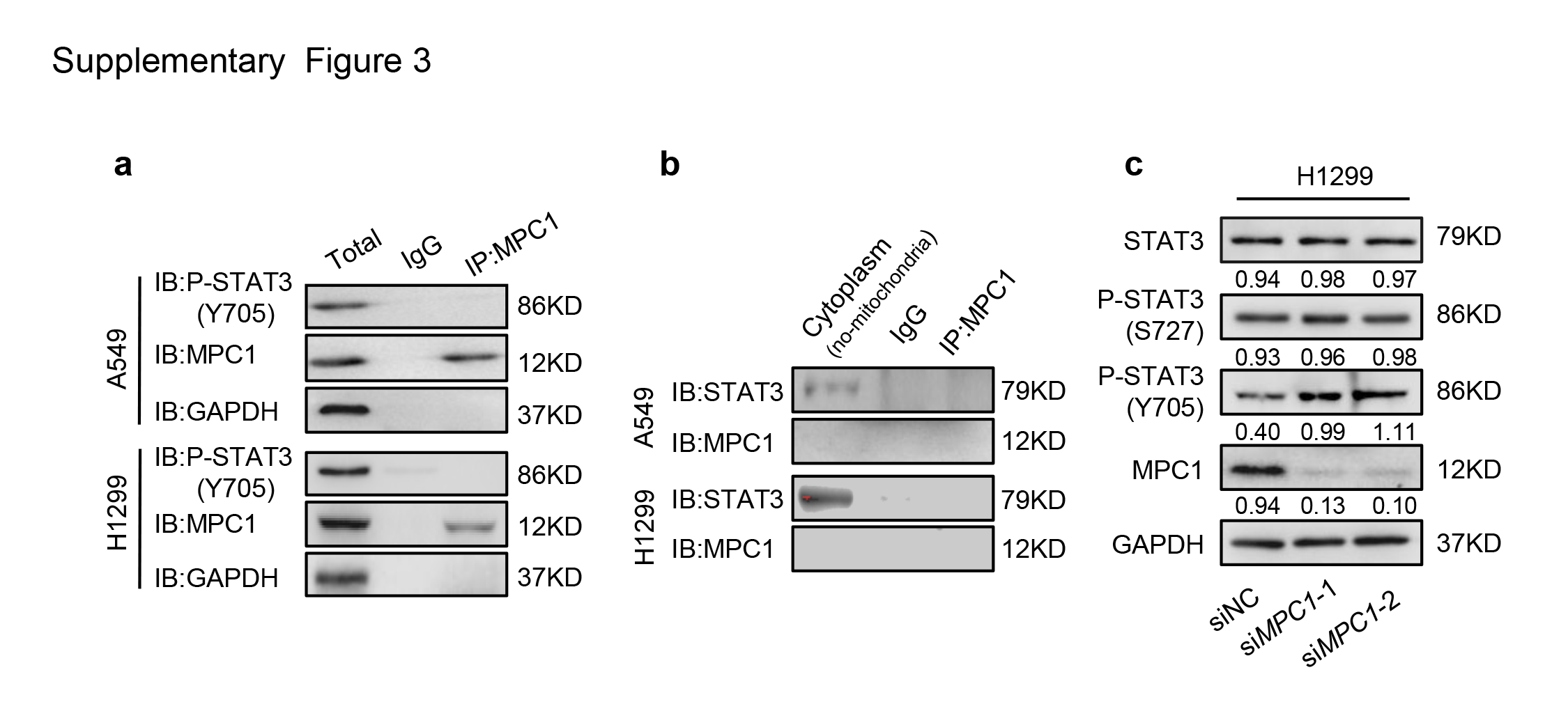

Supplement: Supplementary file 3 — Supplementary Figure 3 [file 41419_2019_1324_MOESM3_ESM.tif]
