# Supplementary material for: MPC1 deficiency accelerates lung adenocarcinoma progression through the STAT3 pathway
Source: Cell Death Dis. 2019 Feb 15;10(3):148. doi: 10.1038/s41419-019-1324-8 (PMC6377639; doi:10.1038/s41419-019-1324-8)
Supplement: Supplementary file 4 — Supplementary Table 1 [file 41419_2019_1324_MOESM4_ESM.doc]

**Supplementary Table 1. The clinical features of the LAC specimens.**

|  |  | WHO Grade |  |
| --- | --- | --- | --- |
| Feature | Ⅰ (n=6) | Ⅱ (n=42) | Ⅲ (n=30) |
| Gender |  |  |  |
| Male | 4 | 18 | 20 |
| Female | 2 | 24 | 10 |
| Age at diagnosis |  |  |  |
| ＜60 | 4 | 15 | 9 |
| ≥60 | 2 | 27 | 21 |
| Location |  |  |  |
| Left lung | 1 | 16 | 9 |
| Right lung | 5 | 26 | 21 |
| TNM stage |  |  |  |
| I | 4 | 21 | 6 |
| II  III | 2  0 | 9  12 | 11  13 |
| T stage |  |  |  |
| T1-T2 | 6 | 35 | 11 |
| T3-T4 | 0 | 7 | 19 |
| EGFR mutation |  |  |  |
| Yes | 1 | 11 | 7 |
| NO | 5 | 31 | 23 |
