# Supplementary material for: MPC1 deficiency accelerates lung adenocarcinoma progression through the STAT3 pathway
Source: Cell Death Dis. 2019 Feb 15;10(3):148. doi: 10.1038/s41419-019-1324-8 (PMC6377639; doi:10.1038/s41419-019-1324-8)
Supplement: Supplementary file 5 — Supplementary Table 2 [file 41419_2019_1324_MOESM5_ESM.doc]

**Supplementary Table 2. The relationship between MPC1 expression and clinicopathological features of LAC patients (n=78).**

|  | MPC1 | |  |
| --- | --- | --- | --- |
| Feature | High (n=44) | Low (n=34) | *P* value |
| Gender |  |  | *P*=0.751 |
| Male | 23 | 19 |  |
| Female | 21 | 15 |  |
| Age at diagnosis |  |  | *P*=0.393 |
| ＜60 | 14 | 14 |  |
| ≥60 | 30 | 20 |  |
| Location |  |  | *P*=0.258 |
| Left lung | 17 | 9 |  |
| Right lung | 27 | 25 |  |
| T stage |  |  | *P*<0.001 |
| T_1-2_ | 39 | 9 |  |
| T_3-4_ | 5 | 25 |  |
| TNM |  |  | *P*=0.013 |
| I | 35 | 18 |  |
| Π+III | 9 | 16 |  |
| Histological grade |  |  | *P*=0.628 |
| Well | 4 | 2 |  |
| Moderate | 23 | 19 |  |
| Poor | 17 | 13 |  |
| EGFR mutation |  |  | *P*=0.304 |
| Yes | 8 | 11 |  |
| NO | 32 | 23 |  |
