# Supplementary material for: MPC1 deficiency accelerates lung adenocarcinoma progression through the STAT3 pathway
Source: Cell Death Dis. 2019 Feb 15;10(3):148. doi: 10.1038/s41419-019-1324-8 (PMC6377639; doi:10.1038/s41419-019-1324-8)
Supplement: Supplementary file 6 — Figure legend [file 41419_2019_1324_MOESM6_ESM.doc]

**Supplementary Figure 1. knockdown and overexpression of MPC1 in LAC cells.**

qRT-PCR showing the mRNA levels of MPC1 in A549 and H1299 cells transfected with overexpression lentivirus and control lentivirus, and in H1299 transfected with knockdown and control vector.

**Supplementary Figure 2. The expression of MMPs proteins in LAC cells after overexpression or knockdown of MPC1.**

**a** Western blotting showing the expression levels of MMP2, MMP3, and MMP7 in LAC cells after overexpression of MPC1. **b** Western blotting showing the expression levels of MMP2, MMP3, and MMP7 in LAC cells after knockdown of MPC1.

**Supplementary Figure 3. MPC1 interacts with STAT3 in LAC cells.**

**a** Co-IP assays showing MPC1 not interacted with P-STAT3(Y705) in LAC MPC1-overexpression cells. **b** Co-IP assays showing MPC1 not interacted with STAT3 in cytoplasm which removed mitochondria in LAC MPC1-overexpression cells. **c** Western blotting showing the expression levels of STAT3, P-STAT3(S727), P-STAT3(Y705) and MPC1 in H1299 cells after knockdown MPC1.

**Supplementary Figure 4. The activation of STAT3 increased the invasion and migration capabilities of OE*MPC1* LAC cells**.

Transwell invasion and migration assays showing the motiled cell after IL-6(100ng/ml) treated OE*MPC1* LAC cells. Scare bar = 50μm.
